# Organic Memristor‐Based Flexible Neural Networks with Bio‐Realistic Synaptic Plasticity for Complex Combinatorial Optimization

**DOI:** 10.1002/advs.202300659

**Published:** 2023-05-15

**Authors:** Hyeongwook Kim, Miseong Kim, Aejin Lee, Hea‐Lim Park, Jaewon Jang, Jin‐Hyuk Bae, In Man Kang, Eun‐Sol Kim, Sin‐Hyung Lee

**Affiliations:** ^1^ School of Electronics Engineering, and School of Electronic and Electrical Engineering Kyungpook National University 80 Daehak‐ro, Buk‐gu Daegu 702‐701 Republic of Korea; ^2^ Department of Materials Science and Engineering Seoul National University of Science and Technology Seoul 01811 Republic of Korea; ^3^ Department of Computer Science Hanyang University 222 Wangsimni‐ro, Seongdong‐gu Seoul 04763 Republic of Korea

**Keywords:** artificial synapse, combinatorial optimization, flexible neural network, organic memristor, synaptic plasticity

## Abstract

Hardware neural networks with mechanical flexibility are promising next‐generation computing systems for smart wearable electronics. Several studies have been conducted on flexible neural networks for practical applications; however, developing systems with complete synaptic plasticity for combinatorial optimization remains challenging. In this study, the metal‐ion injection density is explored as a diffusive parameter of the conductive filament in organic memristors. Additionally, a flexible artificial synapse with bio‐realistic synaptic plasticity is developed using organic memristors that have systematically engineered metal‐ion injections, for the first time. In the proposed artificial synapse, short‐term plasticity (STP), long‐term plasticity, and homeostatic plasticity are independently achieved and are analogous to their biological counterparts. The time windows of the STP and homeostatic plasticity are controlled by the ion‐injection density and electric‐signal conditions, respectively. Moreover, stable capabilities for complex combinatorial optimization in the developed synapse arrays are demonstrated under spike‐dependent operations. This effective concept for realizing flexible neuromorphic systems for complex combinatorial optimization is an essential building block for achieving a new paradigm of wearable smart electronics associated with artificial intelligent systems.

## Graphical abstract

Flexible hardware neural networks for complex combinatorial optimization are demonstrated utilizing the organic memristor‐based artificial synapses. In the proposed synapse, metal‐ion injections are systematically engineered, which leads to the bio‐realistic synaptic plasticity. The developed systems are reliably trained and computed for combinatorial optimization such as the complex max‐cut problems.

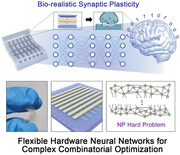

## 1 Introduction

Hardware neural networks with mechanical flexibility have received considerable attention as next‐generation computing systems for smart wearable electronics.^[^ ^1^, ^2^, ^3^ ^]^ A hardware neural network, which is suitable for the parallel computation of large amounts of data, is a promising candidate for overcoming the von Neumann bottleneck.^[^ ^4^, ^5^ ^]^ In such systems, an electric signal is simultaneously processed with the transmission along the synaptic cells, which leads to rapid computation and high energy efficiency. It is essential to develop artificial synapses with bio‐realistic functions to realize practical neuromorphic systems that are similar to their biological counterparts.^[^ ^6^, ^7^, ^8^ ^]^ In the human brain, synapses possess volatile and nonvolatile memory characteristics, called short‐term plasticity (STP) and long‐term plasticity (LTP), respectively, and history‐dependent learning from sequential stimuli is achieved via the correlation of STP and LTP.^[^ ^9^, ^10^ ^]^ Another transient memory feature is homeostatic plasticity, which is induced by hormonal effects. It is combined with synaptic plasticity, which enables the solving of nonpolynomial hard problems that require combinatorial optimization.^[^ ^11^ ^]^ Until now, several studies were conducted to develop flexible and rigid artificial synaptic devices for mimicking STP and LTP.^[^ ^12^, ^13^, ^14^, ^15^, ^16^ ^]^ However, such devices were used only as an analog memory component in neuromorphic systems for simple pattern recognition, due to their restricted functions of synaptic plasticity. To realize practical hardware neural networks capable of solving complex problems, such as combinatorial optimization, it is essential not only to achieve synaptic plasticity (STP and LTP) for computation but also hormone‐based homeostatic plasticity for system stabilization.^[^ ^11^ ^]^ Specifically, in complex neural networks for combinatorial optimization, the signals transmitting from the synaptic cell should possess the decaying noise to obtain the high solution accuracy. Despite considerable effort has been made to achieve the artificial synapse with complete synaptic plasticity, it is still challenging to realize the bio‐realistic synaptic cells.

Solution‐processed organic memristors are favorable for flexible neuromorphic electronics and smart wearable systems owing to their advantages of mechanical flexibility, biocompatibility, and integration density.^[^ ^12^, ^17^, ^18^, ^19^ ^]^ For such devices, nanosized conductive filaments (CFs) are grown or disrupted by the electrochemical metallization (ECM) mechanism, which leads to the resistive switching characteristics of the devices. Moreover, volatile memory characteristics, which are analogous to the STP of synapses, can be induced when the CFs are grown incompletely in devices.^[^ ^18^, ^20^ ^]^ However, it is difficult to manipulate CF dynamics owing to their stochastic and abrupt properties. Therefore, the synaptic plasticity of organic memristors is primarily governed by the inherent properties of the material.^[^ ^21^, ^22^ ^]^ Furthermore, both short‐ and long‐term memory characteristics are governed by a single mechanism for CF growth, which results in difficulties in implementing STP and LTP independently in devices.^[^ ^23^, ^24^ ^]^ Recently, polymer molecular weight has been reported as a diffusive parameter of CFs for replicating synaptic plasticity in organic memristors.^[^ ^25^ ^]^ The memory volatility of the organic memristor changed according to the molecular weight; however, the engineering of the material parameter related to its insulating properties inevitably caused high operating voltages when replicating synaptic plasticity. Therefore, achieving flexible neural networks, which are ideal for smart wearable systems, requires a new versatile parameter for controlling CF dynamics and developing an organic memristor with bio‐realistic synaptic plasticity.

**Figure 1:**
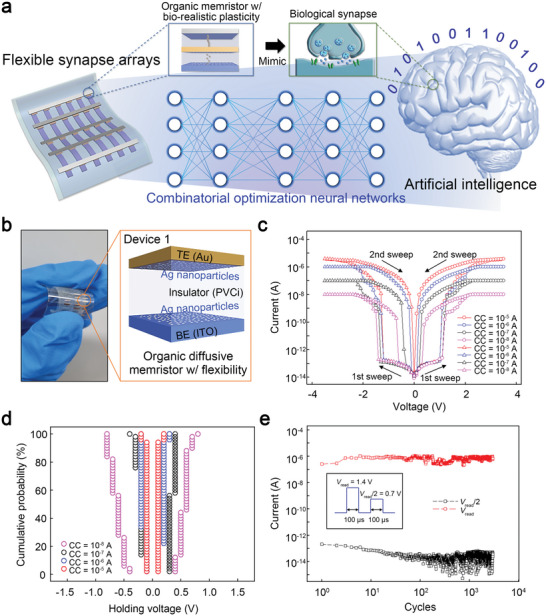
Concepts of flexible neural networks for combinatorial optimization and electrical properties of the organic memristor with limited ion injection. a) A schematic of the organic memristor‐based artificial synapse and complex neural networks for combinatorial optimization. b) A photograph of the developed flexible diffusive memristor with the metal nanoparticles at the interfaces (Device 1). The inset image presents the device configuration. c) Current–voltage features of the device. d) Distributions of the holding voltages of the device measured for the sequential cycles at the compliance currents of 10^−8^, 10^−7^, 10^−6^, and 10^−5^ A. e) The volatile resistive switching performances of the device measured under the repeated pulses (3000 cycles). The voltage pulses of 1.4 and 0.7 V were used for reading the low‐ and high‐resistance states, respectively.

In this study, we demonstrate a flexible artificial synapse with bio‐realistic synaptic plasticity for complex neural networks (see **Figure** **1**a), for the first time. The ion density for the ECM phenomena in the organic memristor was explored as a diffusive parameter for the CF, and it was controlled by active metal (Ag) nanoparticles inserted at the interfaces. As the ion injection during the writing process was restricted, the CF stability degraded, which enhanced the short‐term memory characteristics of the devices. To leverage the independent memory characteristics for STP and LTP, we developed a flexible artificial synapse composed of two different memristor parts (computation and memory parts for STP and LTP respectively), where deficient and sufficient ion injection can be induced at the electric stimulus, respectively. In the proposed flexible synapse, the spike‐dependent learning processes in biological systems were completely mimicked, and the time window of synaptic plasticity was easily tuned via the interfacial Ag particle density for ion injection. In addition, homeostatic plasticity was effectively represented in the device owing to the precisely matched conductance states between the computation and memory parts. Flexible neural networks consisting of the developed synapse arrays were reliably trained and computed for combinatorial optimization, and our network exhibited strong potential for realizing complex systems, such as stochastic Hopfield neural networks.

## 2 Results and Discussion

We prepared a device with Ag nanoparticles on its top and bottom interfaces, Device 1 (see Figure 1b), to study the effects of restricted ion injection during the writing process on the memory characteristics of the organic memristors. The nanoparticles were produced using the Ostwald‐ripening‐assisted self‐assembly method^[^ ^26^, ^27^ ^]^ (see Figure S1, Supporting Information). We utilized inert metals, i.e., gold and indium‐tin‐oxide, as the top and bottom electrodes, respectively, for the ion injection confined at the interfacial Ag particles. A poly(vinyl cinnamate) polymer medium was used as an insulating layer for the stable ECM of the organic memristor.^[^ ^3^, ^18^ ^]^ Note that the top Au electrode was deposited at room temperature (27 °C) to avoid the formation of an Ag–Au alloy.^[^ ^27^, ^28^, ^29^ ^]^ Figure 1c shows the electrical properties of Device 1. The device was first initialized using the electroforming process (see Figure S2, Supporting Information),^[^ ^30^, ^31^ ^]^ and the current–voltage (*I*–*V*) curves were investigated at four different compliance current (CC) values (10^−8^, 10^−7^, 10^−6^, and 10^−5^ A) in an ambient condition (with a relative humidity (RH) of 30%, at 27 °C). In all the CC conditions, the device exhibited volatile threshold‐switching characteristics, and at CC = 10^−5^ A, the switching current level was saturated to a value (≈3.8 **×** 10^−6^ A) lower than the CC value, which indicates self‐compliance current characteristics. This implies that the introduction of Ag nanoparticles for restricted ion injection effectively suppresses CF growth and induces volatile memory characteristics. In addition, the device was stably operated, irrespective of the voltage polarity, owing to the formation of Ag nanoparticles at both the top and bottom interfaces (see Figure S3, Supporting Information). The device also showed the same behaviors in harsh environments (with RH of 50%, at 60 °C), as shown in Figure S4 (Supporting Information). Note that the chemical durability of the organic device can be highly improved through the passivation process.^[^ ^32^ ^]^ We confirmed the electrical characteristics of Device 1 by measuring the threshold‐switching voltages (*V*_th_) and holding voltages (*V*_hold_) of the device for 50 cycles at each CC value. The *V*_th_ value did not change with CC, and only small fluctuations were observed in the switching‐voltage distributions during repeated cycles (see Figure S5, Supporting Information). In typical ECM devices, the *V*_th_ value for resistive switching is not dependent on the CC when the CF is not completely formed.^[^ ^33^ ^]^ In addition, CF is grown in a random fashion, which can lead to variations in the switching voltages during the repeated resistive switching operations of the device.^[^ ^26^, ^34^ ^]^ Figure 1d shows the dispersion of *V*_hold_ under different CC conditions. As the CC value used to adjust the ion injection increased, the CF stability was improved, and *V*_hold_, which maintains the CF structure, was reduced. This means that the control of the metal‐ion injection is directly related to the CF stability in organic memristors. It should be noted that, in the device with the Ag nanoparticles (Device 1), the operating voltages (threshold and holding voltages) were increased with increasing polymer thickness (see Figure S6, Supporting Information), which is consistent with the typical organic ECM devices.^[^ ^35^ ^]^ Additionally, we investigated the cell‐to‐cell uniformity of the device, as shown in Figure S7 (Supporting Information). The spatial variation values, the ratio of the standard deviation to the average value of the postive and negative threshold switching voltages, were ≈0.12 and 0.13 respectively, which are similar to the typical ECM memristors.^[^ ^26^, ^34^ ^]^ Reliable pulse operation is essential for utilizing a volatile organic memristor as a diffusive device in practical neural networks.^[^ ^36^ ^]^ As shown in Figure 1e, we tested the volatile resistive‐switching performance of Device 1 in pulse mode. Voltage pulses of 1.4 and 0.7 V were used to read the low‐ and high‐resistance states of the device, respectively, and the width of each pulse was 100 µs. The device exhibited reliable switching behavior for 3 **×** 10^3^ cycles, and a threshold switching ratio of ≈10^7^ was sustained.

**Figure 2:**
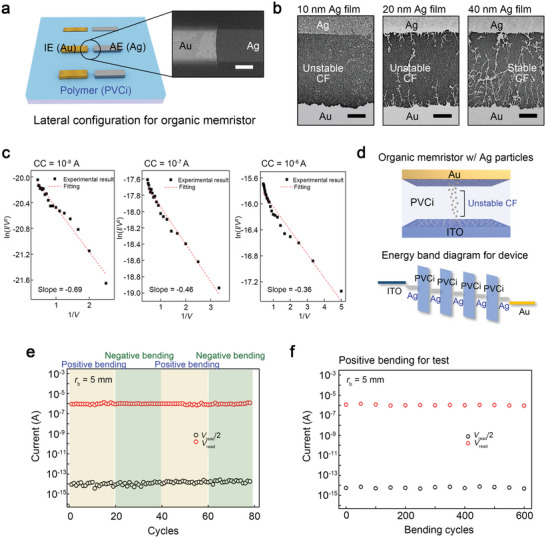
Operating principles and mechanical flexibility of the organic diffusive memristor. a) A schematic presenting lateral‐type devices for analyzing the conduction mechanism of the organic memristor with restricted ion injection. The inset image shows the device structure investigated by the field‐emission scanning electron microscope (FE‐SEM) (scale bar, 100 µm). b) The conductive nanofilament growth in lateral‐type memristors with different thicknesses for the active electrode (10 nm, 20 nm, and 40 nm). The active region of each device was investigated utilizing the FE‐SEM (scale bar, 100 nm). c) The ln(*I*/*V* ^2^) versus 1/*V* curves of the vertical‐type organic memristor with interfacial Ag nanoparticles (Device 1). The graphs were replotted from Figure 1c, which was measured at the positive switching regions. d) A schematic of the operating principle of Device 1. e) Repeated volatile resistive switching behaviors of Device 1 under the positive and negative bending states. f) Mechanical endurance characteristics of Device 1.

We now discuss the operating principle of an organic memristor consisting of interfacial Ag nanoparticles. Three different organic memristors with a lateral configuration were prepared (see **Figure** **2**a) to explore the dynamics of CF growth at the restricted ion injection, and the electrical characteristics of the devices were investigated. Ag and Au were used as the active and inert electrodes of the devices, respectively, and the ion injection in each device was simply set through the active electrode thickness (10, 20, and 40 nm). Note that in the ECM memristor, less ion injection is achieved during the writing process as the active electrode thickness decreases,^[^ ^37^ ^]^ and the electrode thickness can cause degradation of the CF stability, if the gap between the electrodes is significantly high.^[^ ^27^ ^]^ All the devices showed resistive switching behavior under voltage‐sweep measurements (see Figure S8, Supporting Information). However, nonvolatile memory characteristics were achieved only in the device with a 40‐nm thick Ag electrode, which means that the density of the ion injection governs the memory volatility in the organic memristors. We analyzed the conduction mechanism of the devices to estimate the CF structure in each device (see Figure S9, Supporting Information) in the low‐resistance state. Charge transport was achieved for the nonvolatile memristor with a 40‐nm thick Ag film via ohmic conduction, which is indicative of the complete formation of the CF. In contrast, a negative linear dependence of ln(*I*/*V* ^2^) on 1/*V* was clearly observed for devices with volatile memory characteristics, which indicates a tunneling mechanism.^[^ ^38^ ^]^ The tunneling‐barrier width evaluated by the absolute slope value^[^ ^39^ ^]^ was reduced with a decrease in the ion‐injection density. This means that the current flow for volatile devices in the low‐resistance state (LRS) is dominated by the incompletely formed CF, and the thickness of the unstable CF can be effectively tuned by the density of the metal‐ion injection. We then directly investigated the CF growth in lateral‐type memristors using a field‐emission scanning electron microscope, as shown in Figure 2b. As the active electrode for the metal‐ion injection became thicker, a more stable CF was formed after the writing process. The CF grew completely when the 40‐nm thick electrode was used, which is consistent with the conduction mechanisms for the devices, as confirmed in Figure S9 (Supporting Information). Additionally, only small metal islands were investigated for the device with a 20‐nm thick Ag film, which indicates a CF rupture. It should be noted that in ECM memristors, the self‐dissolution phenomenon for the CF is promoted when the CF is incompletely formed.^[^ ^18^ ^]^

We analyzed the conduction mechanism of the vertical‐type organic memristor with interfacial Ag nanoparticles (Device 1) by replotting the ln(*I*/*V* ^2^)–1/*V* curves from the positive switching regions in Figure 1c. In Device 1, the current flow at the LRS followed the tunneling conduction of the unstable CF, and the CF thickness represented by the tunnel‐barrier width was effectively controlled by the CC value. These results are accordance with those of the lateral‐type memristors with the incomplete CF (the devices consisting of the 20‐ or 40‐nm thick Ag electrode). Figure 2d shows the operating principle of the organic memristor. In the device with Ag nanoparticles, the CF was formed incompletely after the writing process, owing to the limited ion injection, and the charges were transported along the unstable CF. Moreover, the immature CF was disrupted by the self‐diffusion of the metal atoms when the electric stimulus was removed, which resulted in the volatile memory characteristics of the device. Two devices with different Ag‐nanoparticle distributions (Devices 2 and 3) were additionally fabricated (see Figure S10, Supporting Information) to further study the relationship between the ion‐injection density and memory volatility in the vertical‐type device. As shown in Figure S11 (Supporting Information), the size and density of the metal particles were adjusted to tune the metal‐ion injection in the devices. In these devices, the volatile memory characteristics evaluated by the *V*_hold_ value were observed to be effectively controlled by the density of the ion injection (see Figure S12, Supporting Information). This implies that the Ag‐particle distribution in Device 1 can be used as a diffusive parameter of the CF to achieve an organic diffusive memristor.

The mechanical flexibility of the device is an essential feature for the development of the synaptic components of wearable neural networks.^[^ ^40^ ^]^ We tested the mechanical flexibility of the vertical‐type organic memristor that consists of interfacial Ag nanoparticles (Device 1), as shown in Figure 2d,e, and Figure S13 (Supporting Information). Under successive bending stresses, the organic memristor exhibited stable threshold resistive‐switching behavior without any degradation in its conductance, regardless of a bending radius. In addition, the device operated stably when subjected to 600 cycles of mechanical stress, thereby exhibiting high mechanical endurance.

**Figure 3:**
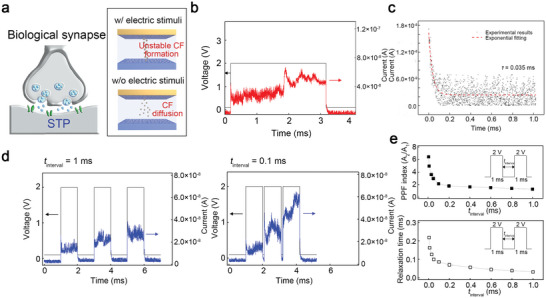
Short‐term plasticity (STP) replicated in the organic memristor with interfacial Ag nanoparticles (Device 1). a) A concept image for STP demonstrated in the device. b) A transient response of Device 1 under a 2‐V voltage. c) The relaxation of the conductance of Device 1 is analyzed in Figure 3b. The experimental result was fitted using an exponential decay function. d) Paired‐pulse facilitation (PPF) of Device 1 by two sequential 2‐V voltage pulses with a width of 1 ms. e) PPF index and relaxation time as a function of the time interval between the two sequential voltage pulses.

We evaluate the capability of Device 1 as an organic diffusive memristor for mimicking the STP of a biological synapse by investigating the dynamic responses of the device under electric stimuli (see **Figure** **3**a). Figure 3b presents the transient current values of the device for a voltage pulse. A 3‐ms voltage pulse of 2 V and a DC bias of 0.1 V were used as an electric stimulus and reading voltage, respectively. When the pulse was applied to the device, the conductance was switched to the LRS from the high‐resistance state (HRS), and it was relaxed back to the HRS as the pulse was removed, thereby indicating the volatile memory characteristics of the device. The relaxation behavior of the device followed an exponential decay function, which is consistent with the STP characteristics of the synapses^[^ ^41^, ^42^ ^]^ (see Figure 3c). Developing an effective strategy to match the time windows of synaptic devices with other neuromorphic components with diverse operating frequencies is important for practical neuromorphic systems.^[^ ^25^, ^43^ ^]^ In our device, the relaxation parameter *τ* in the exponential fitting function was effectively tuned by the distributions of the Ag nanoparticles for ion injection (see Figure 3c; Figure S14, Supporting Information), which implies that the proposed device structure and metal particles can be utilized for developing artificial synapses with diverse time windows. To confirm the computational capability of Device 1, its transient responses to successive electric stimuli were measured, as shown in Figure 3d. Two types of consecutive pulses with different time interval (*t*_interval_) values (1 and 0.1 ms) were applied to the device. The amplitude and width of each pulse were 2 V and 1 ms, respectively. In both measurements, the excitatory post‐synaptic current (EPSC), which is the peak current, increased with an increase in the pulse number, and the EPSC at the last pulse was higher in the case with the shorter *t*_interval_. This result is analogous to paired‐pulse facilitation (PPF), which is a critical STP property in synapses.^[^ ^44^, ^45^ ^]^ This suggests that the growth and diffusion of immature CFs in the device resemble the dynamics of calcium ions in biological systems. Figure 3e shows the PPF index according to *t*_interval_ between electric stimuli. Two consecutive voltage pulses with the same conditions as those of the pulse in Figure 3d were used, and the index value was defined as the ratio between the EPSC values of the first and second pulses. The PPF index increased from 1.29 to 6.42 when *t*_interval_ was varied from 1 ms to 5 µs. Moreover, the relaxation time required for switching back to the HRS in the device also changed from 32 to 218 µs, based on the interval value. This indicates the STP characteristics of the device (see Figure 3e).^[^ ^44^, ^45^ ^]^ As *t*_interval_ between the stimuli increased, the instability of the CF is enhanced owing to the longer time required for the lateral diffusion of the CF, which results in a lower PPF index and a shorter relaxation time in the device. Based on the short‐term memory features demonstrated in Figure 3, Device 1 can be considered a diffusive memristor, which is ideal for demonstrating the bio‐realistic STP in flexible neural networks.

**Figure 4:**
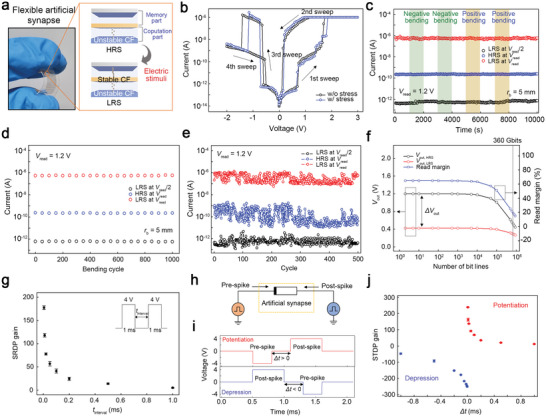
A flexible artificial synapse with spike‐dependent learning capabilities. a) A photograph of a developed flexible artificial synapse with spike‐dependent learning capabilities. The inset image illustrates the operating principle of the device. b) Current–voltage properties of the device measured with and without the bending stress. The bending radius (*r*_b_) for the mechanical stress was 5 mm. c) The retention performance of the device under a flat state and mechanical bending stresses with *r*_b_ = 5 mm. The resistance state in the labels refers to that of the memory part in the synapse. d) Mechanical and e) electrical endurance characteristics of the device. The resistance state in the labels refers to that of the memory part in the synapse. f) The calculated read margin of the synapse cell at the floating scheme. A sensing resistor of 3 MΩ and a reading voltage of 1.2 V were used. g) Spike‐rate‐dependent plasticity of the device. h) The device connected with the two pulse sources acting as neurons to implement spike‐timing‐dependent plasticity (STDP). i) The electric stimuli for pre‐ and post‐synaptic spikes in STDP. j) STDP of the device.

We realized a flexible artificial synapse with complete synaptic plasticity by adjusting the metal‐ion injection in organic memristors, as shown in **Figure** **4**a. The developed artificial synapse comprised two parts with different ion injections (memory and computation) to achieve STP and LTP independently. For the computation part, the ion injection was restricted by inserting interfacial metal nanoparticles instead of an active electrode, as in Device 1. In contrast, a typical memristor structure for nonvolatile memory characteristics (see Figure S15, Supporting Information) was utilized for the memory part. To initialize the device, we performed the electroforming process^[^ ^30^, ^31^ ^]^ (see Figure S16, Supporting Information). Figure 4b shows the *I*–*V* characteristics of the synapse with and without mechanical bending stress. Because the resistance at the HRS of the computation part is larger than that of the memory part, volatile and nonvolatile resistive switching phenomena were achieved sequentially, and the nonvolatile resistance state of the device was governed by the conductance of the memory part in the device. The device operated stably as a reversible memory device with selective characteristics. It should be noted that a diffusive memristor with volatile memory properties can be used as a selector for memory systems with a high integration density by matching the operating voltage and current levels of the device with that of a memory cell.^[^ ^33^, ^46^ ^]^ During the repeated 50 cycles that consisted of voltage sweeps for writing and erasing, the nonvolatile conductance of the device was stably switched from the HRS (or the LRS) to the LRS (or the HRS) (see Figure S17a, Supporting Information). A reading voltage pulse of 1.2 V with a width of 100 µs was used. To confirm the conductance state of the memory part in our flexible synapse, it is necessary to use a reading voltage that is higher than the threshold value (0.8 V) for the transient CF growth in the computational part. The temporal fluctuations, defined as the ratio of the standard deviation to the average value of the writing and erasing voltages, were ≈0.11 and 0.14, respectively, which are comparable with those of conventional ECM memristors.^[^ ^12^, ^47^, ^48^ ^]^ Additionally, eight different synapse cells prepared on a flexible substrate exhibited similar switching voltages (see Figure S17b, Supporting Information). Note that the cell‐to‐cell uniformity and reliability of ECM memristors can be effectively enhanced by localizing CF growth.^[^ ^18^, ^49^ ^]^

We performed retention and cycle tests to specifically evaluate the electrical and mechanical durability performance of the synapse (see Figure 4c–e). In the retention test, repeated positive and negative bending deformations with a radius (*r*_b_) of 5 mm were applied to the device for 4 × 10^3^ s, as shown in Figure 4c. Our device exhibited stable nonvolatile memory states and selective characteristics for 10^4^ s, regardless of the tensile and compressive stresses. The current on/off ratio at a reading voltage (*V*_read_) of 1.2 V was ≈10^4^. Moreover, the selectivity, defined as the ratio of the current value at *V*_read_ = 1.2 V to that at *V*_read_/2 = 0.6 V, was ≈10^6^ in the device, which is considerably superior to the other synaptic cells with selective behaviors.^[^ ^30^, ^50^, ^51^, ^52^, ^53^ ^]^ Note that when a voltage of *V*_read_/2 = 0.6 V, which is not high enough to induce the transient CF growth in the computation part, is applied to the device, the current flow is suppressed regardless of the resistance state of the memory part. A bending cycle test was then performed to confirm the mechanical durability of the device, as shown in Figure 4d. Each cycle consisted of positive and negative bending stresses with *r*_b_ = 5 mm. For 10^3^ cycles, the memory and selective performances of the device were stably maintained without any degradation, thereby indicating its strong potential as a component for practical wearable systems. In addition, the devices showed high mechanical durability at the bending stresses with different values (20, 10, and 5 mm) of *r*_b_ (see Figure S18, Supporting Information). Figure 4e presents the electrical endurance performance of the device, which was measured via a cycle test composed of repeated voltage sweeps. Our synapse exhibited reversible switching characteristics and reliable selective performance over 500 cycles, which is comparable to those of inorganic memristors for practical applications.^[^ ^27^, ^54^ ^]^

The development of highly integrated data‐storage systems based on memristors requires memory cells with selective characteristics for suppressing sneak current paths in crossbar‐structured arrays.^[^ ^50^, ^51^, ^52^, ^53^ ^]^ We conducted a numerical analysis to estimate the potential of the developed synapse as a memory component in complex systems (see Figure S19, Supporting Information). As shown in Figure 4f, the crossbar array consisting of our artificial synapses possessed an integration density larger than 360 Gbit in the floating scheme, which is considered to be an outstanding performance compared to previous studies.^[^ ^12^, ^55^ ^]^ Another essential feature of synapse devices for practical memory applications is stable pulse operation.^[^ ^56^ ^]^ The resistive switching characteristics of the synapse under pulse conditions were investigated, as shown in Figure S20 (Supporting Information). For the switching processes (writing and erasing), voltage pulses of 5 V and ‐3 V were applied to the device, respectively. The conductance of the device changed stably in the pulse mode, and selective behavior was clearly observed. The writing and erasing times of the device were ≈44 and 23 µs, respectively. Note that in ECM memristors, the switching times for CF growth and rupture can be simply decreased by optimizing the voltage amplitude.^[^ ^57^, ^58^ ^]^

We now discuss the spike‐dependent learning capability of our synapse to realize bio‐realistic neural networks. History‐dependent learning rules in the human brain, spike‐rate‐dependent plasticity (SRDP), and spike‐timing‐dependent plasticity (STDP) have been demonstrated in developed synapses. In the developed device, the interaction between the memory and computation parts induces a spike‐dependent switching operation (see Figure S21, Supporting Information). When electric stimuli are applied to the device sequentially, the first stimulation focuses on the computation part with a relatively high resistance at the initial state, which leads to the growth of an unstable CF for the STP. However, the distribution of the next pulse at each part is governed by *t*_interval_. Specifically, when a shorter value of *t*_interval_ is used, the time required for the CF diffusion decreases in the computation part, which results in a higher conductance in the computation part and relatively larger voltage in the memory part under the second pulse. This synaptic cell with the history‐dependent learning capability leads to the simple and efficient operating scheme of the hardware neural networks, because it can be trained without complex engineering of the input signal pulses. Note that, in the systems consisting of the typical memristors, the complex processes including the pulse overlap are externally required in the learning processes.^[^ ^25^, ^43^ ^]^ Figure 4g shows the SRDP features that were measured using the device. The device was stimulated by two pulses of 4 V with a width of 1 ms, and the *t*_interval_ value between the pulses was adjusted from 1 ms to 5 µs. The SRDP gain, which is defined as the ratio of the varied conductance value to the initial value, was increased from 4.0 to 181.4 as *t*_interval_ decreased. STDP is a critical property of biological systems in controlling synaptic connections, in which the synaptic weight is potentiated (or depressed) according to the time difference (Δ*t*) between the pre‐ and post‐synaptic spikes.^[^ ^59^ ^]^ We applied pre‐ and post‐synaptic spikes to the device to measure the STDP characteristics of the developed synapse (see Figure 4h). Simple electric stimuli, similar to those of biological systems, were utilized as synaptic spikes (see **Figure** **4i**). As shown in Figure 4j, our device stably reflected STDP. Potentiation and depression of the device conductance were achieved selectively, depending on the temporal sequence of the spikes, and the STDP gain estimated by the ratio of the conductance change to the initial conductance was effectively controlled by Δ*t*. This indicates that the developed device can act as a synaptic cell for achieving hardware‐based spiking neural networks with a high energy efficiency. Moreover, hormone‐based homeostatic plasticity was easily replicated in the device during the reading process by engineering the input pulse (see Figure S22, Supporting Information). In our synapse, the computation part based on Device 1 possesses relatively low conductance at the HRS and LRS compared to the memory part; thus, the current level in the reading process is dominated by the computation part with short‐term memory features. To mimic homeostatic plasticity in the device, we utilized a reading pulse comprising two regions: the first region induces the transient memory effect, and the second region reads the synaptic conductance. When we apply the reading pulse to the device, the resistance of the computation part can be transiently reduced by the first region of the reading pulse, and it leads to the decaying current level for the second region of the reading pulse. Although the current fluctuations were slightly observed due to the accuracy limits of the pulse measuring system,^[^ ^25^, ^36^ ^]^ a transient increase in the conductance of the device, which is analogous to homeostatic plasticity,^[^ ^11^, ^60^ ^]^ was successfully controlled by the amplitude of the first region of the reading stimulus. In the hardware neural networks, homeostatic plasticity in the synaptic cell can be utilized for delicate signal processing to solve the complex problems with high accuracy.^[^ ^11^ ^]^ Based on the superior synaptic characteristics and the high integration density, compared to the other devices^[^ ^61^, ^62^, ^63^, ^64^ ^]^ (see Table S1, Supporting Information), it can be considered that our developed flexible artificial synapse is highly promising for smart wearable electronics.

**Figure 5:**
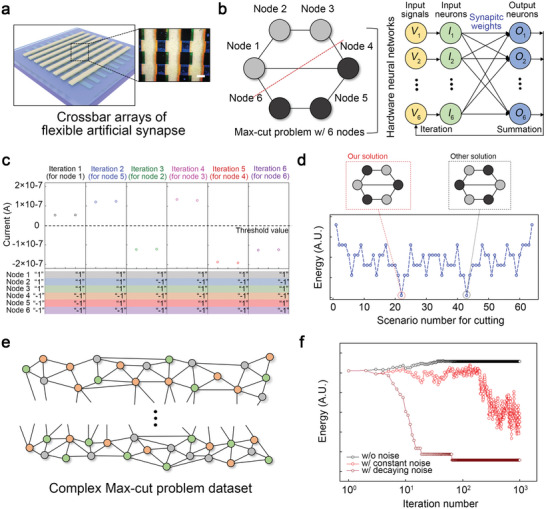
Hardware neural networks based on the developed synapse for combinatorial optimization. a) A schematic of the crossbar arrays of the developed artificial synapse. The inset shows a microscopic image of the fabricated synapse arrays (scale bar, 500 µm). b) Hopfield neural network developed for solving the max‐cut problem composed of six nodes. c) Iteration processes for combinatorial optimization of the max‐cut problem. d) Hopfield energy values of all the scenarios for the max‐cut problem. Inset images present the optimized solutions for the max‐cut problem. e) Complex graphs consisting of the large number of nodes and complex topologies. f) The Hopfield energy of the neural network systems used to solve the max‐cut problem composed of 100 nodes, based on the synaptic noise conditions.

Using the developed artificial synapse with bio‐realistic synaptic plasticity (STP, LTP, and homeostatic plasticity), we constructed hardware neural networks for combinatorial optimization, as shown in **Figure** 5a. The graph max‐cut problem is a well‐known nonpolynomial hard problem that can be solved through combinatorial optimization.^[^ ^65^, ^66^, ^67^ ^]^ The max‐cut problem is generally used for optimizing complex circuit designs, and its aim is to identify a line that cuts the largest number of edges linking two different nodes in a graph composed of several nodes. The Hopfield neural network (HNN),^[^ ^11^, ^68^ ^]^ a recurrent neural network consisting of neurons (detailed information in Note S1, Supporting Information), was developed using a 6 × 6 array of synapses to solve the max‐cut problem composed of six nodes. Initially, the cutting line for partitioning the graph was set randomly (see Figure 5b), and the values of “1” or “‐1” were selectively matched to the nodes according to the regions defined by the line. All the bit and word lines of the synapse array for the HNN were matched to the nodes, and the synaptic cells of the array were trained by the SRDP‐based learning process (see Figure S23, Supporting Information). As shown in Figure 5c, following the HNN rules, we performed successive iteration processes to update the six node values. In the iteration process for each target node, all bit lines were set to 0 V, and the word lines for the nodes that were not connected to the target node were grounded. By contrast, we applied voltage pulses to the word lines corresponding to the nodes linked to the target. When the node value was “1” (or “−1”), a voltage pulse of 1.0 V (or −1.0 V) was biased to the word line. The current level of the bit line matched to the target node was checked to update the node value. In the case that the current of the bit line that was matched to the target was higher than the threshold value of 0 A, the target node value was set to “1”. Contrarily, the node value was modified as “−1” when the bit line current was lower than 0 A. After the optimization processes, the values of nodes 1, 3, and 5 were “1,” and those of the other nodes were “−1”. For solving the problem, our system consumed ≈75.3 fJ, which is highly superior to other systems.^[^ ^69^ ^]^ We estimated the Hopfield energy values of all scenarios for the max‐cut problem consisting of six nodes to confirm the validity of our optimized solution, as shown in Figure 5d. In combinatorial optimization based on the HNN, the system energy converged to the minimized value while achieving optimization. Our solution identified the minimum energy among the scenarios, thereby indicating a valid optimization for the max‐cut problem. This means that the neural networks based on our synapse possess a strong potential for solving nonpolynomial hard problems with high energy efficiency.

We specifically confirm the potential of the developed synapse arrays for complex combinatorial optimization by performing numerical simulations to achieve a globally optimum solution for more complex graph‐cut problems. A benchmark of the max‐cut problem,^[^ ^60^ ^]^ which consisted of large graphs with nodes ranging from 60 to 100, was utilized in the analysis, and the HNN for combinatorial optimization was constructed using the developed synapse arrays. The synaptic weight of the cell was estimated from the experimental results, as shown in Figure 4. It is well known that the energy landscape of complex graphs (Figure 5e), which consists of a large number of nodes and complex topologies, contains multiple local optima.^[^ ^11^, ^62^ ^]^ Therefore, strategies to move away from local optima, such as simulated annealing or perturbation, are required for complex cases. To resolve this problem, we added synaptic noise based on the homeostatic plasticity during the optimization phase. The exponentially decaying noise represented in our synaptic device was utilized, and the noise amplitude of the device was approximately one‐hundredth of the original signal (see Figure S24, Supporting Information). As shown in the simulation results (see Figure 5f), an appropriate level of noise added to the synaptic weights is crucial for finding the global optimum in the complex max‐cut problem with 100 nodes. Specifically, the HNN that comprises the developed synapse with time‐decaying noise exhibits a robust and efficient optimization performance. This indicates that our synapse arrays with bio‐realistic synaptic plasticity are suitable for complex combinatorial optimizations.

## 3 Conclusion

In conclusion, we implemented flexible neural networks with bio‐realistic synaptic plasticity to solve nonpolynomial hard problems. We demonstrated that the metal‐ion density controlled by interfacial Ag nanoparticles acts as a diffusive parameter of the CF in organic memristors. In these devices, volatile memory characteristics that are analogous to STP were achieved via restricted ion injection and the resultant immature CF growth. In addition, the time window for the STP of the device was effectively tuned by the distribution of Ag particles. This concept of replicating STP in the organic memristors may be extended to the systems consisting of other polymer films and active metal particles. We developed a flexible artificial synapse with complete synaptic plasticity, including STP, LTP, and homeostatic plasticity, by spatially controlling metal‐ion injection in the organic memristor. The developed synapse was composed of two parts with different ion injections to replicate STP and LTP independently. In the computation part with deficient ion injection, an immature CF was formed based on the electric stimuli, which resulted in STP characteristics. However, for the memory part with sufficient ion injection, conductance was governed by mature CF growth, which led to LTP features. Moreover, hormone‐based homeostatic plasticity, which is required for achieving hardware‐based combinatorial optimization, was simply replicated in the device by engineering the reading‐signal pulse. Our flexible synapse exhibited spike‐dependent learning capabilities, including SRDP and STDP, under simple electric stimuli, similar to those of biological systems. Furthermore, the crossbar arrays of the synapse exhibited a high potential for constructing complex neural networks, which are ideal for combinatorial optimization. This promising strategy for developing flexible neural networks that are suitable for solving nonpolynomial hard problems is an essential building block for realizing a new paradigm of wearable smart electronics associated with artificial intelligent systems.

## 4 Experimental Section

The geometric profiles of the devices were measured using a surface profiler (DektakXT‐A, Bruker). The electrical features of the devices were investigated using a semiconductor parameter analyzer (4200‐SCS, Keithley) combined with an ultrafast *I*–*V* module (4225‐PMU, Keithley). A scanning voltage was applied to the top electrode (Au) to measure the electrical performance of the organic memristors, and the bottom electrode (ITO) was grounded. A field‐emission scanning electron microscope (S‐4800, Hitachi) was used to analyze the configuration and CF growth in lateral‐type memristors.

To fabricate a flexible memristor with interfacial Ag nanoparticles (Device 1), an ITO‐patterned polyethylene naphthalate (PEN) substrate was cleaned successively via ultrasonication in acetone, isopropyl alcohol, and deionized water for 30 min. A 0.5‐nm Ag film was thermally deposited on the substrate at 0.1 Å s^−1^ under 10^−6^ Torr to produce Ag nanoparticles at the bottom interface. The particles were then baked at 140 °C for 1 h to induce Ostwald ripening. As an insulating medium, poly(vinyl cinnamate) (PVCi) dissolved in cyclopentanone in 5 wt.% was spin‐coated on the substrate with Ag particles at a rate of 3000 rpm for 30 s. Then, the polymer layer was baked at 130 °C for 2 h to remove any residual solvent. The thickness of the polymer medium was ≈280 nm. The same process used for the bottom interface was used to form Ag nanoparticles at the top interface. For the top electrode, a 50‐nm thick gold film was thermally evaporated at 2 Å s^−1^ under 10^−6^ Torr. The lateral dimensions of the device were 500 µm × 500 µm.

To prepare lateral‐type organic memristors, a glass substrate was successively cleaned via ultrasonication in acetone, isopropyl alcohol, and deionized water for 30 min. As an insulator, the PVCi solution, the same as for Device 1, was spin‐coated on the substrate at a rate of 3000 rpm for 30 s, and the polymer film was annealed at 130 °C for 2 h to remove any residual solvent. A 50‐nm‐thick Au film was deposited on the substrate via thermal evaporation at 1 Å s^−1^ under 10^–6^ Torr for the inert electrode. The inert electrode was then patterned using a fluoropolymer (EGC‐1700, 3M) via typical softlithography and a wet‐etching process using an etchant (TFA, Transene) for Au. As the active electrode, a 10 nm (or 20 and 40 nm) Ag film was thermally deposited on the fluoropolymer‐patterned substrate at 1 Å s^−1^ under 10^–6^ Torr. The active electrode was patterned using a lift‐off process to remove the fluoropolymer. The width of each electrode and the gap between the electrodes were ≈200 µm and 450 nm, respectively.

To fabricate the flexible artificial synapse, an organic memristor with interfacial Ag nanoparticles was first produced as the computation part, and a typical organic memristor was prepared on the computation part for the memory part. The PEN substrate with ITO patterns was sequentially cleaned via ultrasonication in acetone, isopropyl alcohol, and deionized water for 30 min. Ag nanoparticles at the bottom interface of the memory part were formed on the substrate by thermally depositing an Ag film with a thickness of 0.5 nm at 0.1 Å s^−1^ under 10^–6^ Torr. The Ag particles were baked at 140 °C for 1 h to promote Ostwald ripening. A PVCi film with a 280‐nm thickness was prepared as the polymer medium over the substrate by spin‐coating it with PVCi dissolved in 5 wt.% cyclopentanone at a rate of 3000 rpm for 30 s. The polymer film was annealed at 130 °C for 2 h to remove any residual solvents. The same process used for the Ag particles at the bottom interface was utilized to produce those at the top interface. A 50‐nm Au film was thermally deposited on the polymer medium with the Ag particles at the rate of 2.0 Å s^−1^ under 10^−6^ Torr as the top electrode of the computation part and the bottom electrode of the memory part. To prepare the insulating medium for the memory part, PVCi dissolved in 5 wt.% cyclopentanone was spin‐coated on the Au layer at a rate of 2000 rpm for 30 s. Then, the polymer layer was baked at 130 °C for 2 h to remove any residual solvent. The thickness of the polymer medium was ≈320 nm. For the top electrode of the memory part, a 50‐nm Ag film was thermally evaporated on the polymer film at a rate of 0.8 Å s^−1^ under 10^−6^ Torr. The lateral dimensions of the synapses were 500 × 500 µm.

## Conflict of Interest

The authors declare no conflict of interest.

## Author Contributions

H.K., E.‐S.K., and S.‐H.L. initiated and designed the experiments, and analyzed the data. H.K., S.‐H.L., and E.‐S.K. wrote the draft of the manuscript. S.‐H.L. and H.‐L.P. fabricated and characterized the devices. E.‐S.K. performed the numerical simulations for the devices. H.K. and S.‐H.L. measured the electrical characteristics of the devices. M.K., A.L., J.J., J.‐H.B., and I.M.K. assisted in device electrical characterizations and data analysis. S.‐H.L. provided overall supervision of the work. All authors have read and approved the final manuscript.

## Supporting information

Supporting Information

## Data Availability

The data that support the findings of this study are available from the corresponding author upon reasonable request.
