# Supplementary material for: Organic Memristor‐Based Flexible Neural Networks with Bio‐Realistic Synaptic Plasticity for Complex Combinatorial Optimization
Source: Adv Sci (Weinh). 2023 May 15;10(19):2300659. doi: 10.1002/advs.202300659 (PMC10323658; doi:10.1002/advs.202300659)
Supplement: Supplementary file 1 — Supporting Information [file ADVS-10-2300659-s001.pdf]

ADVANCED SCIENCE
Open Access

Supporting Information

for *Adv. Sci.*, DOI 10.1002/advs.202300659

Organic Memristor-Based Flexible Neural Networks with Bio-Realistic Synaptic Plasticity for Complex Combinatorial Optimization

Hyeongwook Kim, Miseong Kim, Aejin Lee, Hea-Lim Park, Jaewon Jang, Jin-Hyuk Bae, In Man Kang, Eun-Sol Kim and Sin-Hyung Lee**

Supporting Information

Organic Memristor-based Flexible Neural Networks with Bio-realistic Synaptic Plasticity for Complex Combinatorial Optimization

Hyeongwook Kim, Miseong Kim, Aejin Lee, Hea-Lim Park, Jaewon Jang, Jin-Hyuk Bae, In Man Kang, Eun-Sol Kim, and Sin-Hyung Lee**

Note S1.

We provide in detailed explanation for graph-cut problems. In this paper, a graph max-cut problem, which is a well-known non-polynomial (NP) hard problem, is solved using Hopfield Neural Network (HNN).

The Hopfield Neural Network (HNN) is one of the recurrent neural networks consists of neurons having activity dynamics with Lyapunov function and synaptic connections between the neurons.

The state value x_i of the neuron V_I at time t is defined as follows:

$$x_i(t) = \begin{cases} 1, if \sum_j w_{ij} x_j(t-1) \geq \theta_i \\ -1, otherwise \end{cases}$$

where w_{ij} is the weight of the connection (synapse) between node (neuron) V_i and V_j. $x_i(t)$ and θ_i represent the state of the node i at time t and the threshold value of the node V_i, respectively.

In this paper, we assume that the state value $x_i \in \{-1,1\}$, which is the discrete-valued HNN.

The graph max-cut problem aims at finding the way to divide the node set V into two subsets S, $V \backslash S$ across maximum edge weights.

Consider an undirected graph $G = (V, E)$ where $V = \{v_1, v_2, \ldots, v_n\}$ and $E = \{(i,j) | i,j \in V \text{ and } i \neq j\}$ represent the vertex and the edge set, respectively.

Here, each edge E_{ij} has a real-valued weight w_{ij}, thus, the weight-valued matrix $W \in \mathbb{R}^{n \times n}$ is called the weight matrix.

For the graph cut problem, we consider a set $\delta(S) = \{(i,j) \in E | i \in S, j \in V\backslash S\}$ which consists of edges contributing to the cut. Formally, the max-cut problem is defined as an optimization problem, which finds the $S \subseteq V$ such that $\max_{S\subseteq V} \sum_{(i,j)\in\delta(S)} w_{ij}$. With assumption about the discrete-valued HNN, it is well-known that the graph max-cut problem is equivalent to the following problem,

$$\max_{x\in\{-1,1\}^n} \sum_{i<j} w_{ij} \frac{1 - x_i x_j}{2} = \frac{1}{4} x^T W' x$$

where $W' = (w'_{ij})$ and $w'_{ij} = -w_{ij}$.

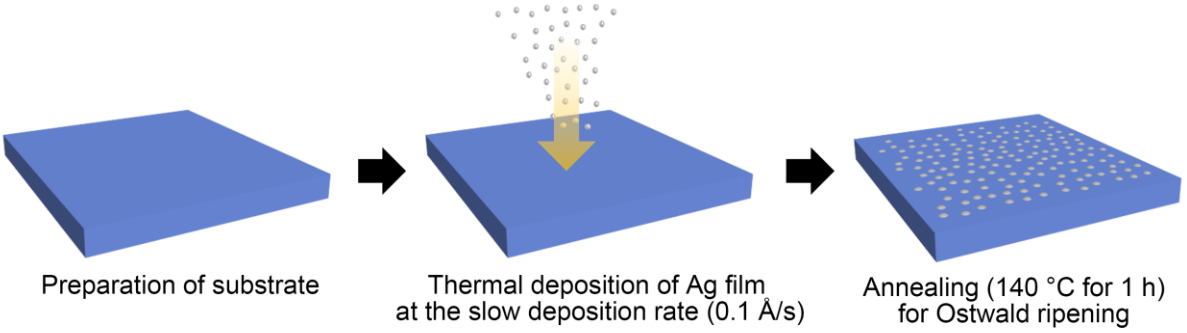

Figure S1. The Ostwald-ripening-assisted self-assembly method for producing the Ag nanoparticles. Since Ag has a surface energy higher than that of ITO or the polymer film, the as-deposited Ag metal at the slow deposition rate tends to ball up on the ITO electrode or the polymer layer. In addition, the Ag clusters formed via the thermal evaporation can be coalesced as a sphere through the annealing process for promoting the Ostwald ripening effect.

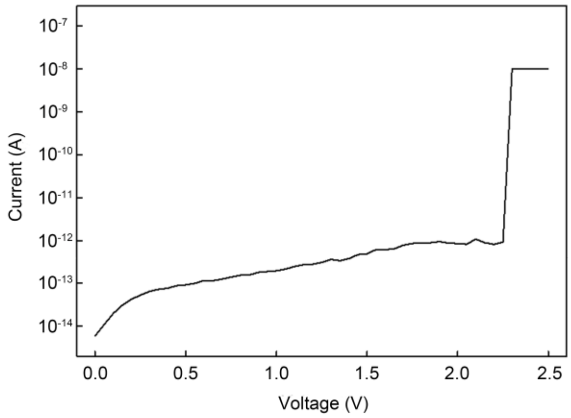

Figure S2. An electroforming process for initializing Device 1.

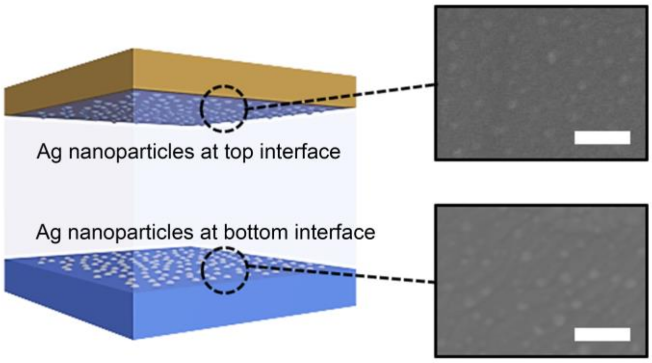

Figure S3. A schematic illustrating the organic memristor with the interfacial Ag nanoparticles (Device 1). Inset image shows the field emission scanning electron microscopy images of the Ag nanoparticles produced at the top and bottom interfaces of the device (scale bar, 140 nm).

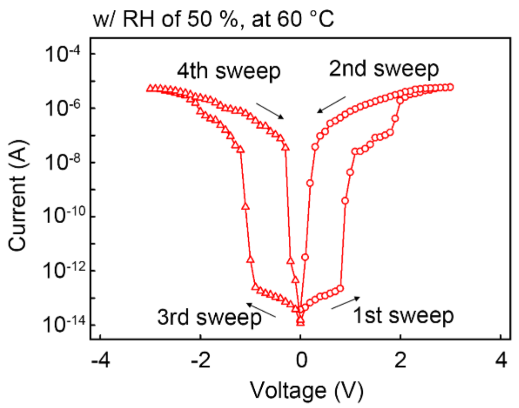

Figure S4. Current-voltage curves of Device 1 measured in harsh environments (with a relative humidity (RH) of 50 %, at 60 °C)

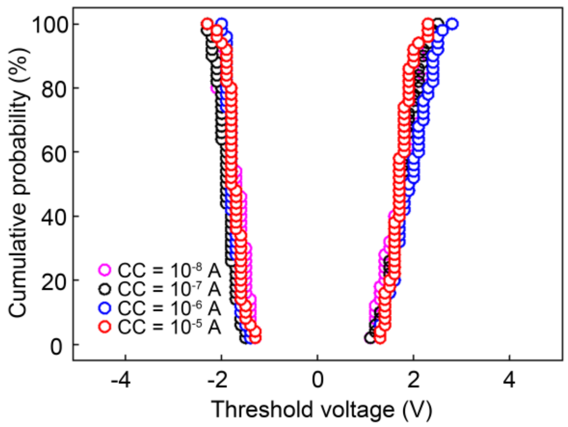

Figure S5. Dispersion of the threshold switching voltages of Device 1 measured during the sequential cycles at the compliance currents of 10^{-8}, 10^{-7}, 10^{-6}, and 10^{-5} A.

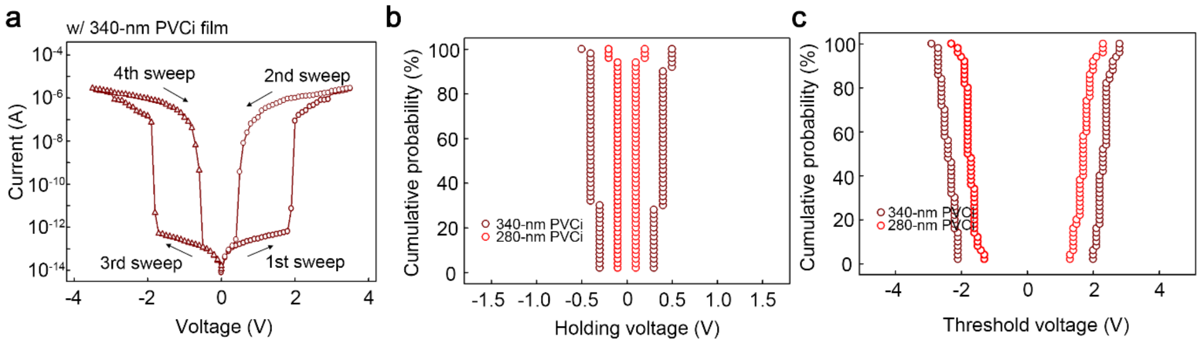

Figure S6. Effects of the polymer thickness on the electrical characteristics of Device 1. (a) *I-V* curves of the device with the 340-nm polymer film. Distributions of (b) the holding and (c) threshold voltages of the devices with different polymer thickness measured for the sequential cycles at the compliance current of 10^{-5} A.

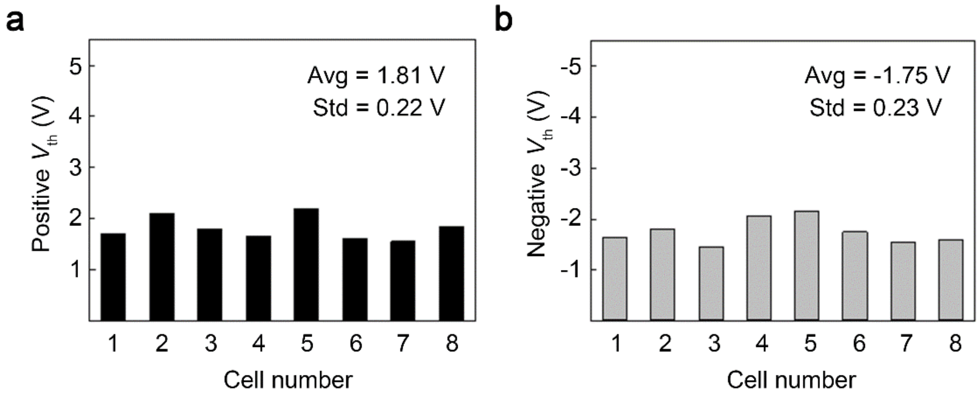

Figure S7. The cell-to-cell uniformity of Device 1. Dispersions of (a) the positive and (b) negative threshold switching voltages of the eight different cells in a single substrate. Each cell was measured at the compliance current of 10^{-5} A.

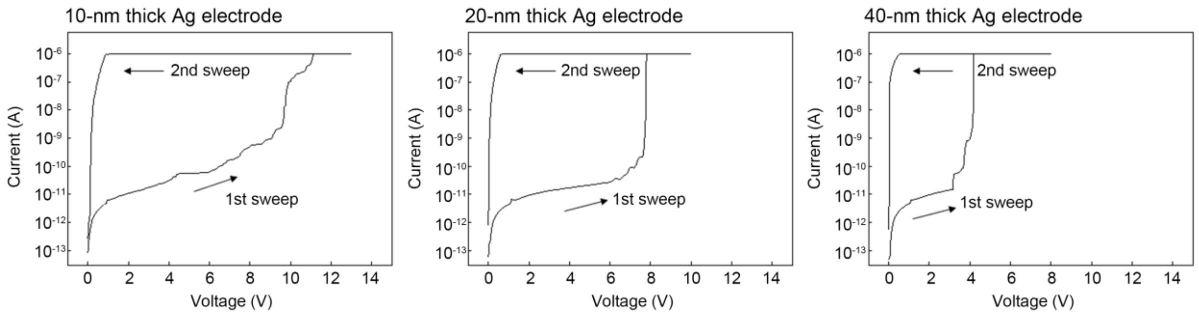

Figure S8. Current–voltage characteristics of the lateral-type organic memristors with the different active electrode (Ag) thickness values.

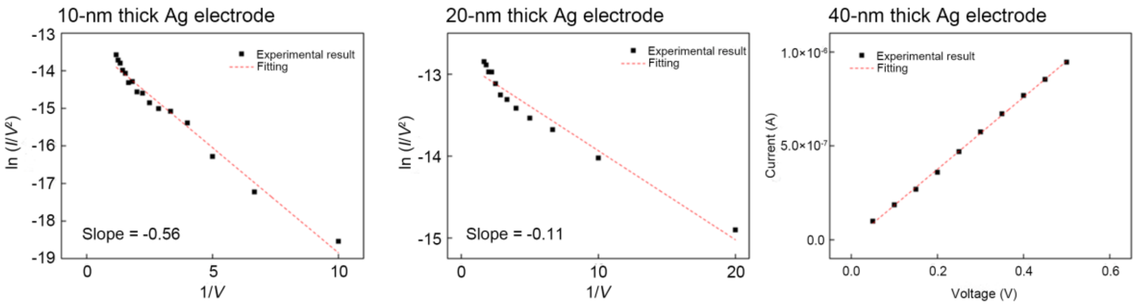

Figure S9. The ln(I/V^2) versus 1/V curves of the lateral-type organic memristors with the different active electrode (Ag) thickness values. The graphs were replotted from in Figure S5, which was measured at the low resistance state.

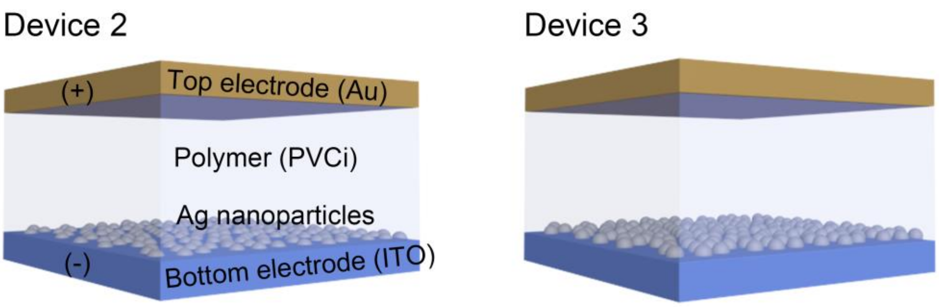

Figure S10. Schematics showing the structures of the vertical-type memristors with the different distributions of the interfacial Ag nanoparticles (Device 2 and Device 3).

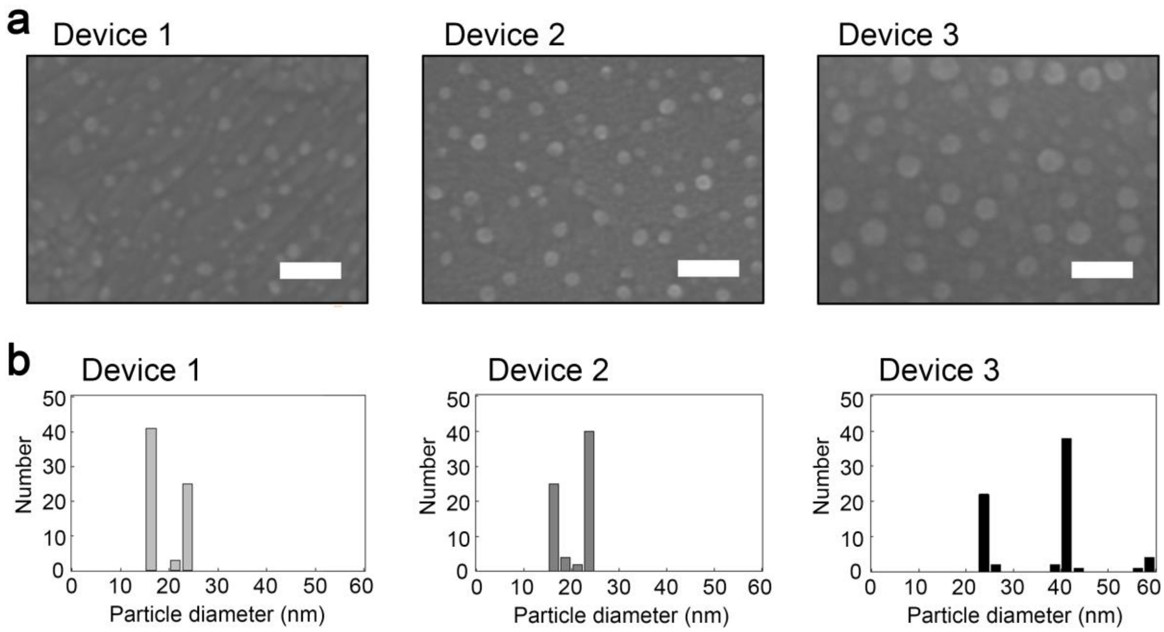

Figure S11. (a) The field emission scanning electron microscopy images for the Ag nanoparticles at the bottom interfaces in the vertical-type organic memristors (Device 1, 2, and 3) (scale bar, 125 nm). (b) Distributions of the Ag nanoparticles at the bottom interfaces in the devices.

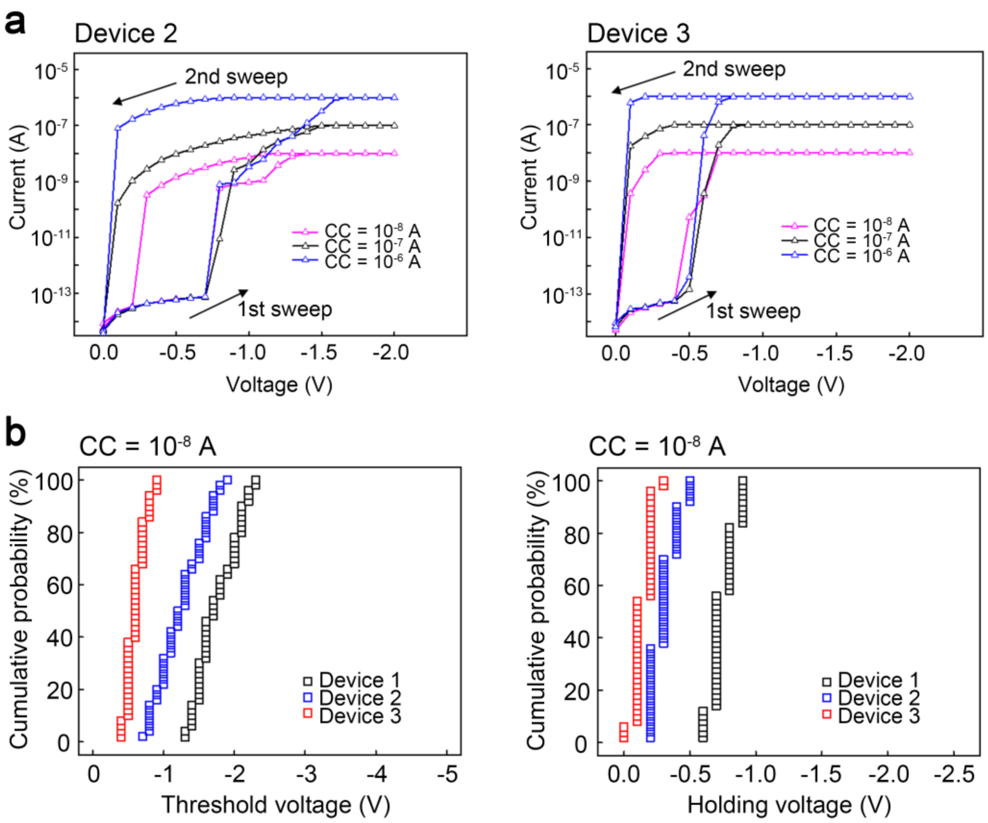

Figure S12. (a) Current–voltage characteristics of Device 2 and Device 3. (b) Distributions of the threshold switching voltages (left) and the holding voltages (right) in the vertical-type organic memristors (Device 1, Device 2, and Device 3) at the compliance current of 10^{-8} A.

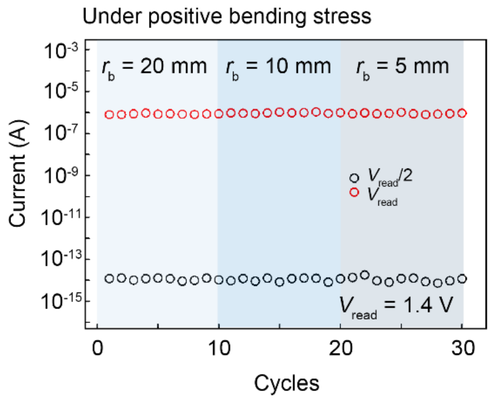

Figure S13. Volatile resistive switching characteristics of Device 1 under the positive bending states with different values of a bending radius (r_b).

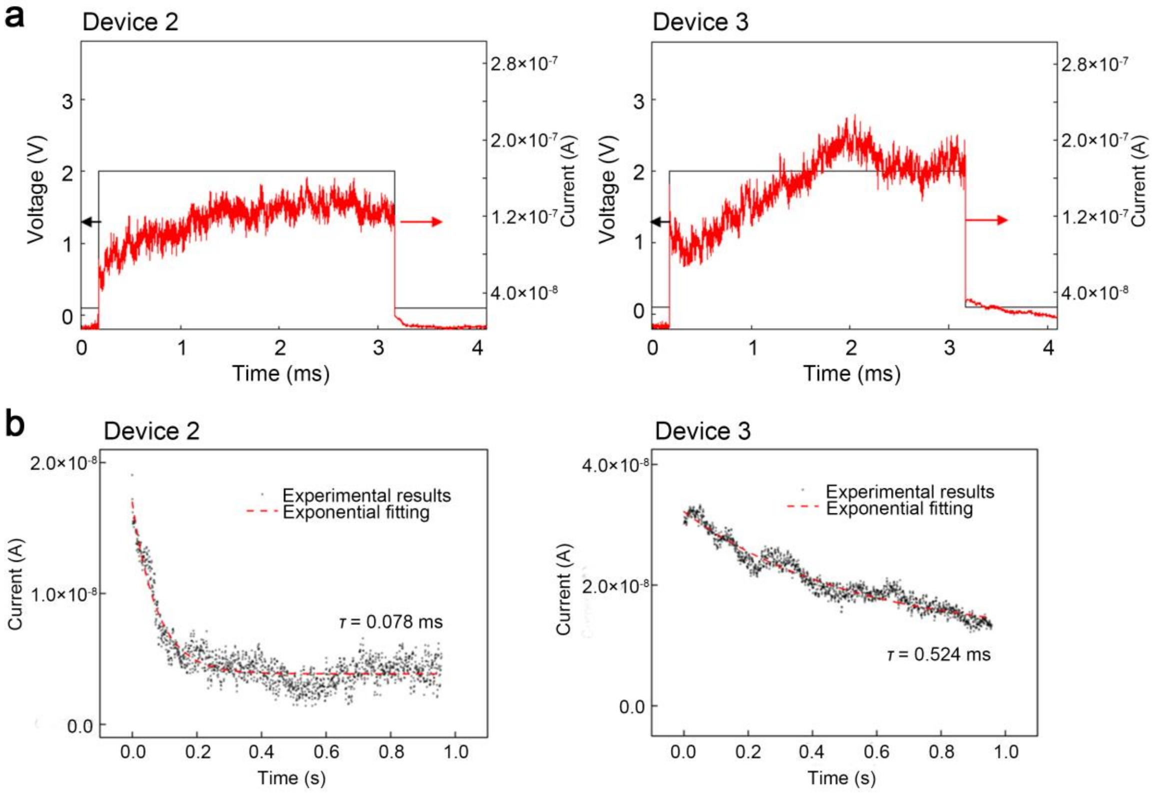

Figure S14. (a) Transient responses of Device 2 and Device 3 under the 2-V voltage. (b) The relaxation feature of each device conductance analyzed in Figure S10a. The experimental results were fitted by exponential decay functions.

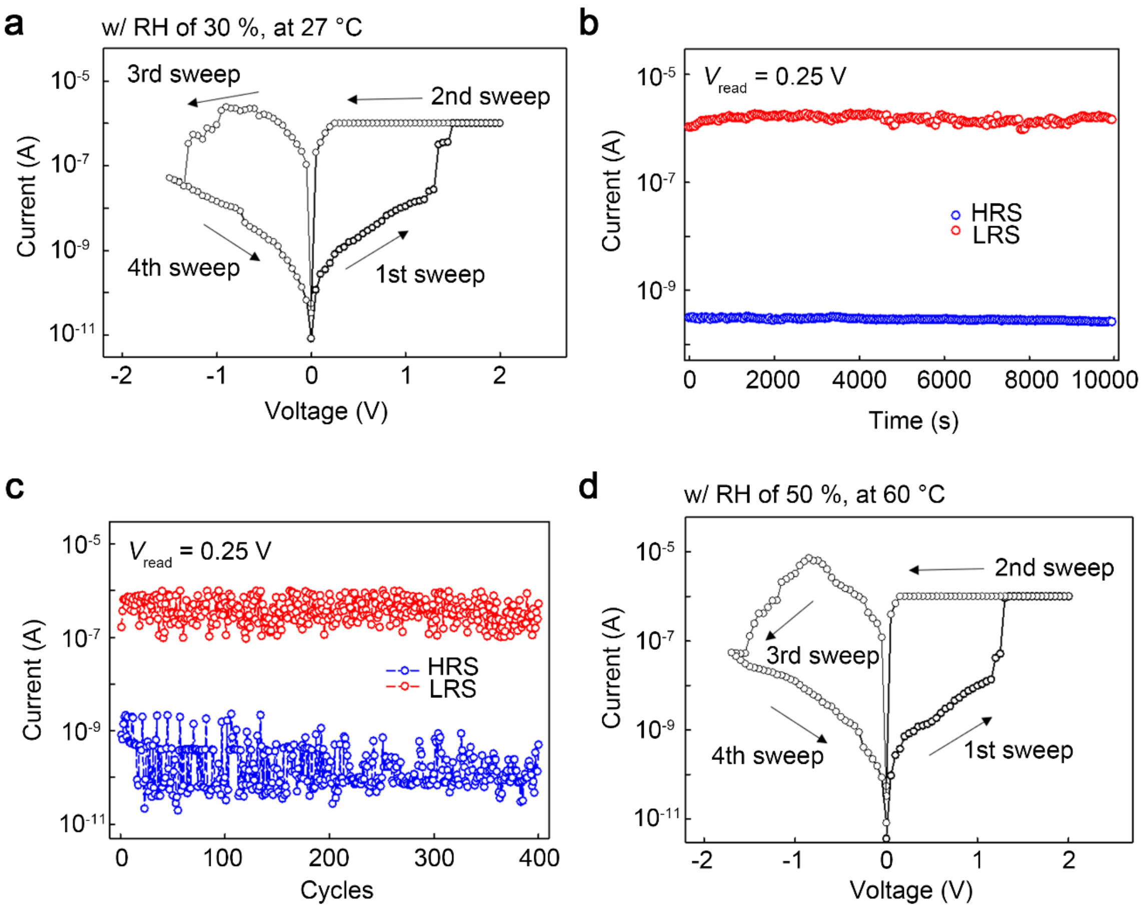

Figure S15. Electrical properties of the conventional organic memristor with the same structure as of the memory part of the developed artificial synapse. (a) Current–voltage characteristics of the device in an ambient condition (with a relative humidity (RH) of 30%, at 27 °C). (b) A retention performance of the device. (c) Electrical endurance characteristics of the device. (d) Current-voltage curves of the device in the harsh environments (with RH of 50 %, at 60 °C).

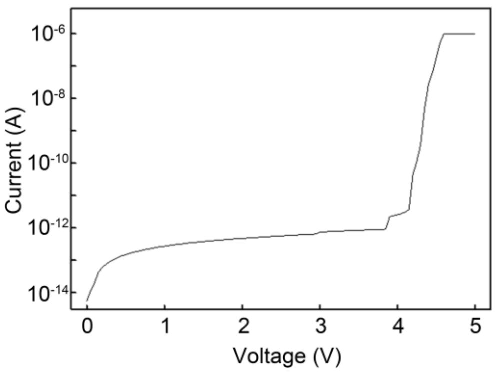

Figure S16. An electroforming process for initializing the artificial synapse.

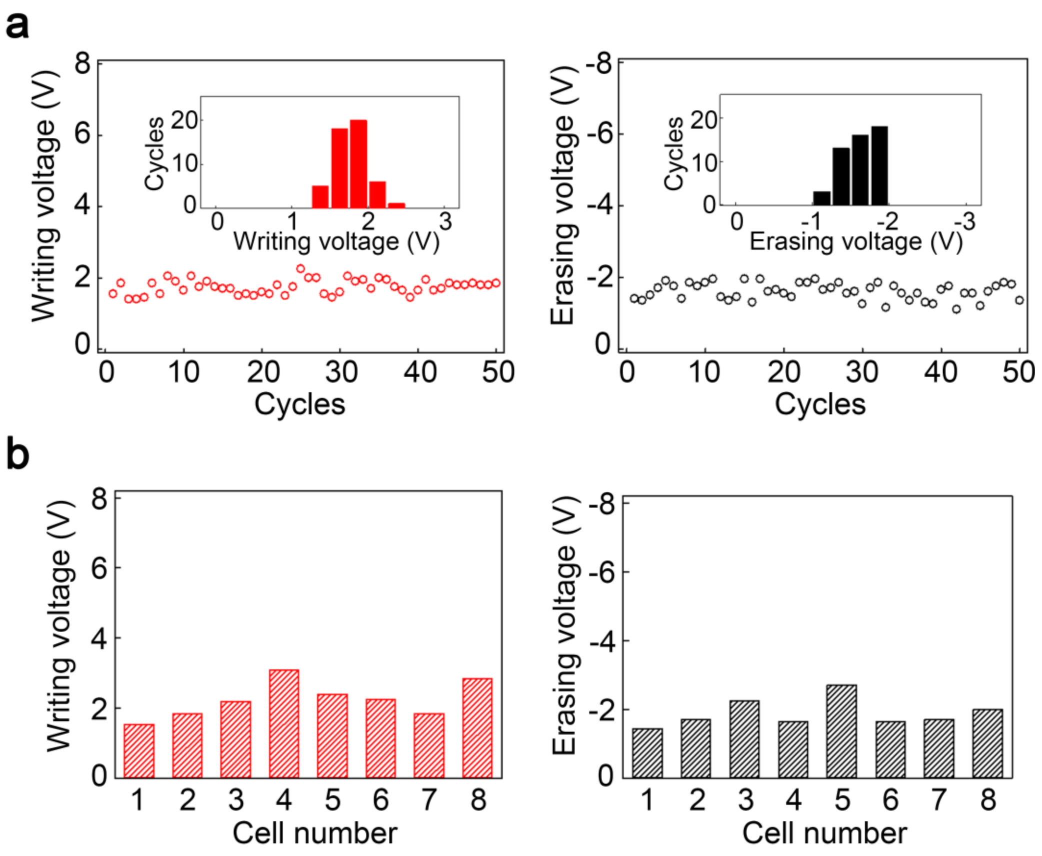

Figure S17. (a) Dispersions of the switching votages measured during the repeated 50 cycles in the developed artificial synapse. (b) Dispersions of the switching voltages measured in eight different synapse cells.

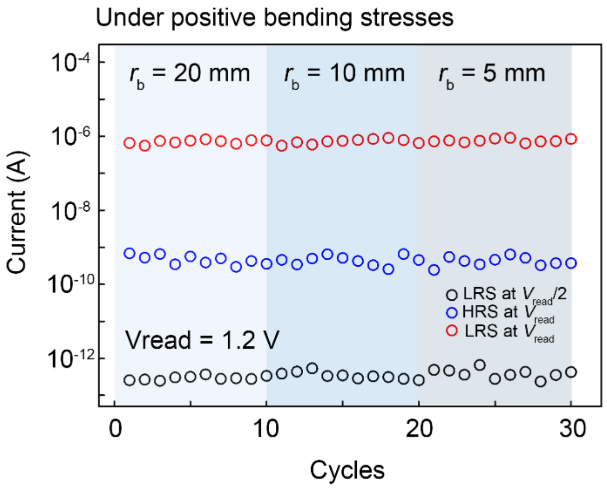

Figure S18. Resistive switching and selective characteristics of the artificial under the positive bending states with different values of a bending radius (r_b).

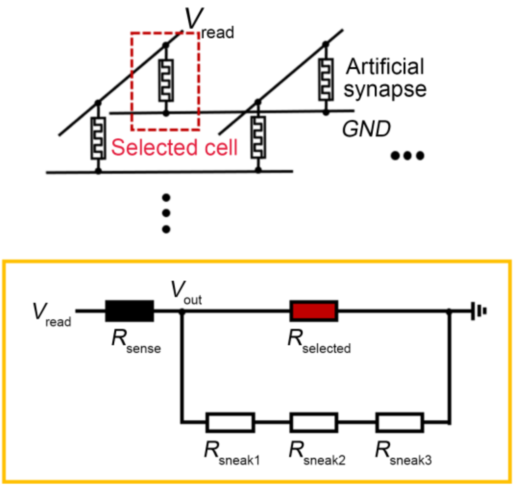

Figure S19. A schematic image showing a circuit for analyzing the read margin according to the number of a bit line in the crossbar arrays consisting of the synapse cell. An inset shows the modeled sneak current paths of the crossbar arrays of the synapse. The read voltage (V_{read}) of 1.2 V, and the sensing resistor (R_{sense}) of 3 MΩ were used.

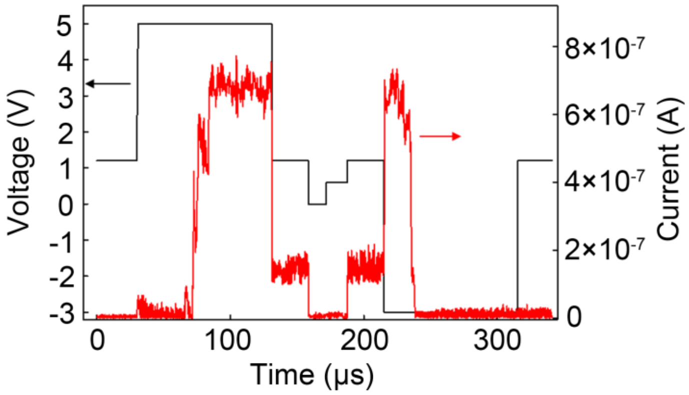

Figure S20. Switching characteristics of the artificial synapse under the pulse mode. For the writing and erasing processes, the voltage pulses with the amplitude values of 5 V and -3V were utilized respectively. The writing and erasing times for the device were about 44 μs and 23 μs respectively.

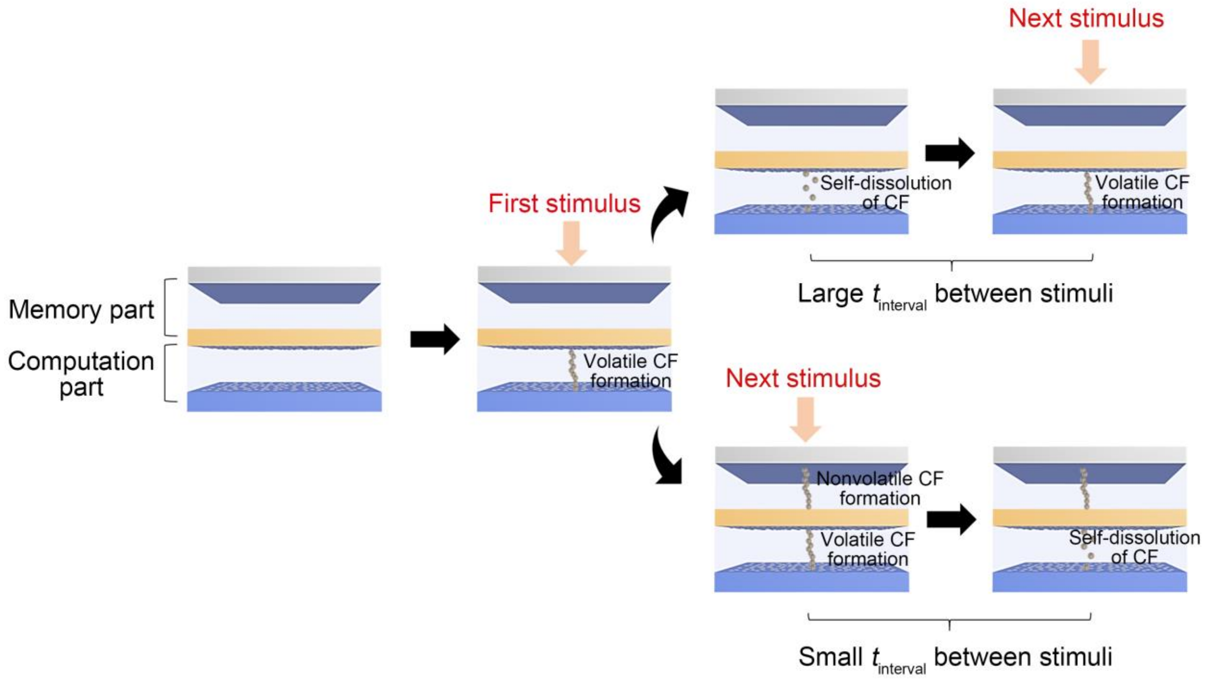

Figure S21. A schematic image showing the interaction of the memory and computation parts in the artificial synapse for the spike-dependent switching operation.

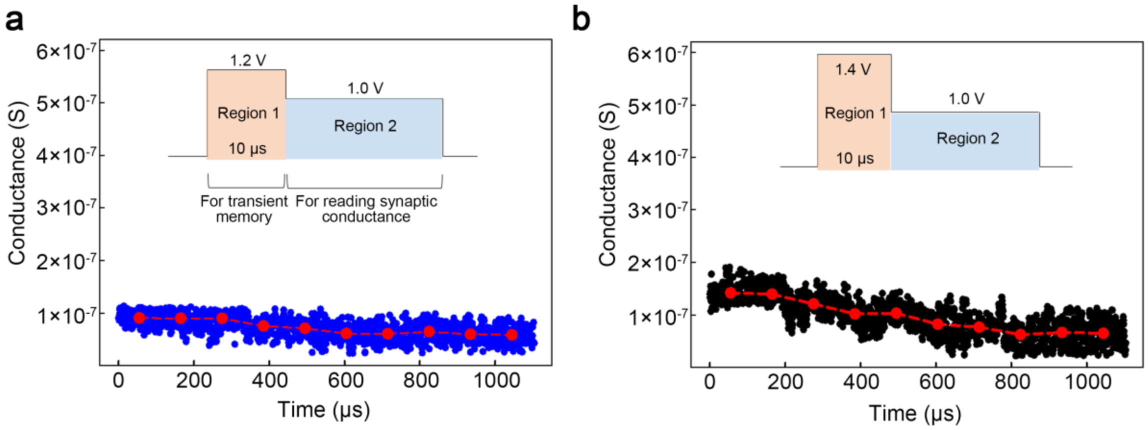

Figure S22. Homeostatic plasticity replicated in the developed synapse device. The reading pulse comprising of the two regions: Region 1 for inspiring the transient memory effect, and Region 2 for reading the synaptic conductance. A transient increase in the conductance of the device by the reading pulses with (a) 1.2-V Region 1 and (b) 1.4-V Region 1, respectively. The slight current fluctuations were also measured owing to the accuracy limits of the pulse measuring system.

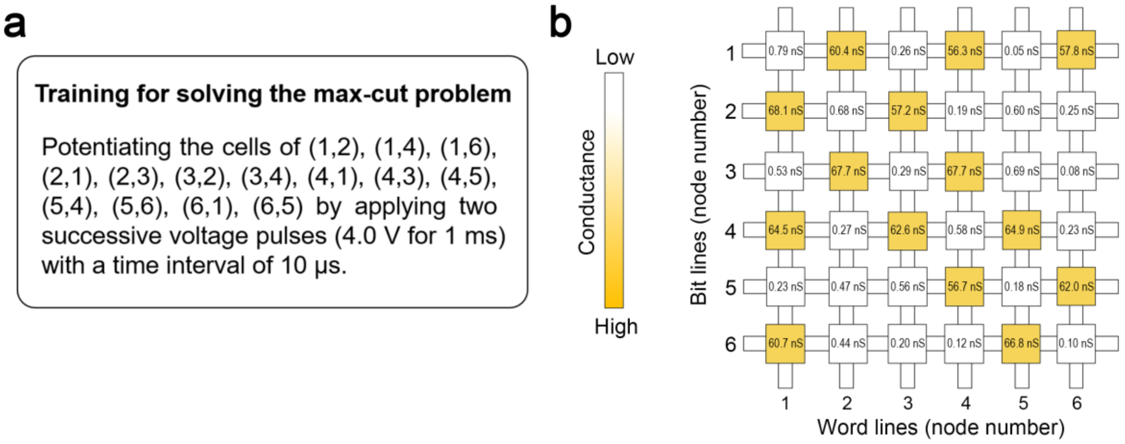

Figure S23. Training of the developed neural networks for solving the max-cut problem consisting of the six nodes. (a) A flow chart for training the synapse arrays. For the training processes, a floating scheme was used. (b) The weight (conductance) distribution of the synapse array after the training processes.

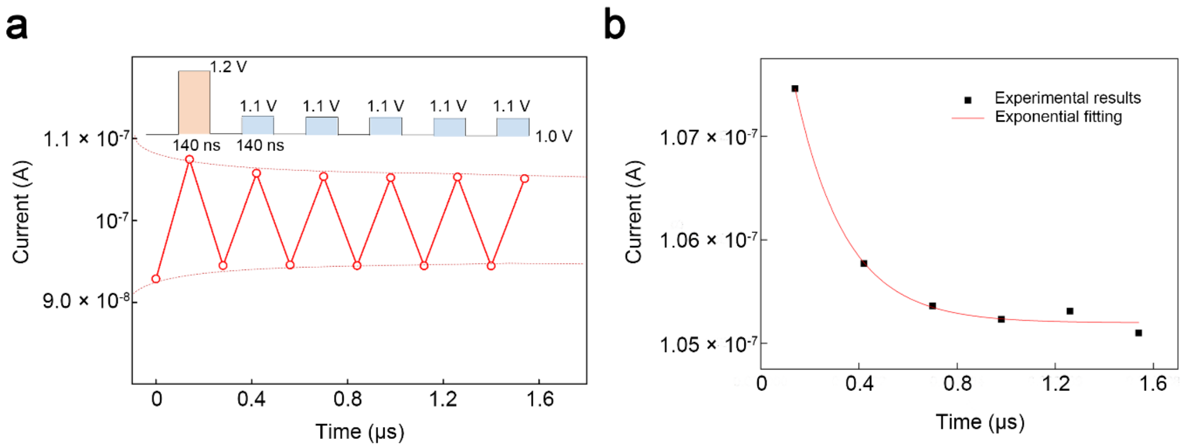

Figure S24. (a) Synaptic noise represented in our flexible synapse. The reading pulse was engineered for achieving the decaying noise. (b) An exponentially decaying characteristics of the synaptic weight (conductance) in the device.

Device structure	Write/Erase voltage	On/off ratio	Selectivity	Mechanical flexibility	Synaptic function	Application	Ref.
Au/P(VDF-TrFE)/NTO	5.0 V / -5.0 V	~ 10^2	-	X	STP/LTP/PPF /STDP (w/ pulse engineering)	-	[10]
Pt/TiO_2/AlO_x/Pt	3.0 V / -1.0 V	~ 10^3	-	O	PPF/STDP (w/ pulse engineering)	Pattern recognition	[13]
Pt/BFO/LSMO	10.0 V / -8.0 V	~ 10	-	O	PPF/STDP (w/ pulse engineering)	-	[15]
Ag/PVCi/ITO	2.0 V / -2.0 V	~ 10^2	-	O	STP/LTP/PPF (w/ pulse engineering)	Pattern recognition	[18]
ITO/PAA/PEI/ITO	-3.0 V / -3.0 V	~ 10	-	O	STP/LTP/PPF /STDP	-	[19]
Ag/PMMA/Ag/ PMMA/ITO	40 V / -20 V	~10	-	X	STP/LTP/PPF /SRDP/STDP	Pattern recognition	[25]
Pt/SiO_xN_y:Ag/Pt/HfO_x/TiN	2.0 V / -2.0 V	~ 10^2	-	X	STP/LTP/PPF /SRDP/STDP	-	[34]
Pt/BFO/LSMO	7.0 V / -6.0 V	~10^2	-	O	STP/LTP/PPF /STDP (w/ pulse engineering)	-	[61]
Ni/ZnO/TiN	1.5 V / -1.5 V	~10	-	X	STP/LTP/PPF	Pattern recognition	[62]
Ag/AgOx//CAAAAKAAAAK//GaOx/EGaIn	-0.4 V / 0.4 V	~10^2	-	X	STP/LTP/PPF /SRDP/STDP	Waveform recognition	[63]
Ag/TiO_2/FTO	3.0 V / -2.5 V	~10^3	-	X	LTP/PPF/STDP	Pattern recognition	[64]
Ag/PVCi/Au/Ag particles/PVCi/Ag particles/ITO	2.0 V / -1.5 V	~ 10^4	~ 10^6	O	STP/LTP/PPF /SRDP/STDP /hormon-based homeostatic plasticity	Combinatorial optimization	This work

Table S1. Comparison of performances of artificial synapses with a 2-terminal structure.
